# Clinicopathologic features of metastatic small cell carcinoma of the prostate to the liver: a series of four cases

**DOI:** 10.1186/s13000-021-01096-1

**Published:** 2021-04-23

**Authors:** Phoenix D. Bell, Aaron R. Huber, Diana Agostini-Vulaj

**Affiliations:** Department of Pathology and Laboratory Medicine, University of Rochester Medical Center, Rochester, NY 14642 USA

**Keywords:** Small cell neuroendocrine carcinoma, Prostate, Liver metastasis, Androgen deprivation therapy, Case series

## Abstract

**Background:**

Small cell neuroendocrine carcinoma of the prostate (SCNECP) is a rare, aggressive subtype of prostate carcinoma. Most SCNECP arise from conventional prostate adenocarcinoma (CPAC) treated with androgen deprivation therapy (ADT).

**Case presentations:**

We identified four cases of CPAC treated with ADT, which evolved to SCNECP with liver metastasis. The average interval between the diagnosis of CPAC and SCNECP was 102 months (range: 12 to 168). Histologically, the tumors showed nests of cells with high nuclear:cytoplasmic ratios, granular chromatin, and frequent mitoses. All cases were synaptophysin, chromogranin, and AE1/AE3 positive, with a Ki-67 labeling index ≥70%. NKX3.1 was negative in all but one case and TTF-1 was positive in half. Weak ERG positivity by IHC was seen in one case which also demonstrated the *TMPRSS2-ERG* gene rearrangement; all other cases were negative for ERG by IHC. Serum prostate specific antigen (PSA) levels were normal to near-normal in all. The median interval between the diagnosis of SCNECP and death was 3.25 months (range: 0.75 to 26).

**Conclusions:**

Our case series highlights the importance of considering a prostate primary, even in the setting of normal PSA levels and loss of prostate markers, when diagnosing neuroendocrine carcinoma in the liver. Further, we emphasize the significance of diagnosing SCNECP that metastasizes to the liver, as it portends a particularly dismal prognosis.

## Background

Small cell neuroendocrine carcinoma of the prostate (SCNECP) is a rare, aggressive type of prostate carcinoma, accounting for 0.5–2% of all prostate malignancies [1]. SCNECP often arises in patients who have undergone androgen deprivation therapy (ADT) for conventional (acinar) prostate adenocarcinoma (CPAC) and who develop disease recurrence (castrate-resistant prostate cancer) [2]. Many patients present with advanced disease, often involving visceral and lytic bony metastasis [2, 3]. Once diagnosed with SCNECP, most patients die within 1 year [4]. Histologically, the diagnosis is based upon morphologic and immunohistochemical (IHC) features; however, metastatic lesions can be challenging, as these they are often associated with low to normal serum PSA levels and negative prostate immunostains [2]. Correct diagnosis of a metastatic lesion involves careful clinical, radiologic, and pathologic correlation. Herein, we present four patients with metastatic SCNECP to the liver, who presented with normal to near-normal PSA levels. Liver biopsies showed NKX3.1 negativity in all but one case, with weak positivity, and TTF-1 positivity in 50% of cases. ERG was weakly positive in one case, this case also demonstrated the *TMPRSS2-ERG* gene rearrangement; all other cases were negative for ERG by IHC. Our case series highlights the importance of considering a prostate primary, even in the setting of normal PSA levels and loss of prostate immunostains, when diagnosing a metastatic neuroendocrine lesion in the liver. Further, we emphasize the importance of correctly diagnosing a SCNECP that metastasizes to the liver, as it portends a particularly dismal prognosis.

## Case presentations

Four patients had a past medical history of CPAC treated with ADT with a subsequent diagnosis of SCNECP. The Gleason Grade (including biopsy or resection) was 3 + 4 = 7 in two patients and 4 + 5 = 9 in two patients. All available H&E slides for prostate biopsy and/or resection cases were reviewed, no evidence of neuroendocrine differentiation were seen in these specimens respectively. The average PSA level at the time of CPAC diagnosis was 32.5 ng/mL (range: 5.6 to 68.9; reference: 0-4 ng/mL). The average interval between the diagnosis of CPAC and SCNECP was 102 months (range: 12 to 168). The average age at the time of SCNECP diagnosis was 73 (range: 68–76). The average PSA level at the time of SCNECP diagnosis was 4.82 ng/mL (range: 0.31 to 8.77; reference: 0-4 ng/mL). The liver site sampled for the diagnosis of SCNEP included right hepatic lobe lesion in 2 cases, while the site was not specified in the remaining 2 cases; in all instances abdominal imaging (computated tomography (CT) or magnetic resonance imaging (MRI)) demonstrated innumerable hepatic lesions. Carcinoembryonic Antigen (CEA) and Carbohydrate Antigen 19–9 (CA19–9) levels were available for 3/4 (75%) and 2/4 (50%) of patients, respectively. The average CEA was 28.4 ng/mL (range: 4.8 to 52.3; reference: 0–4.7 ng/mL) and average CA19–9 was 533 U/mL (range 18–1048; reference: 0-35 U/mL). Treatment modalities employed throughout patients’ courses of disease (CPAC and SCNECP) included: ADT (4/4 patients; 100%), radiation (4/4 patients; 100%), chemotherapy (3/4 patients; 75%), or radical prostatectomy (2/4 patients, 50%). In addition to the liver, sites of metastasis included the rectum, retroperitoneal lymph nodes, adrenal gland, and bone. All patients died as a result of their disease. The median interval between the diagnosis of SCNECP and death was 3.25 months (range: 0.75 to 26 months) (Table 1).

**Table 1:** Clinicopathologic features of metastatic small cell carcinoma of the prostate to the liver

	PATIENT 1	PATIENT 2	PATIENT 3	PATIENT 4
**Description of Cases**				
Age at SCNECP Diagnosis (years)	74	68	74	76
CPAC Gleason Grade	3 + 4 = 7	4 + 5 = 9	3 + 4 = 7	4 + 5 = 9
PSA Level (ng/mL) at CPAC Diagnosis	5.81	50	5.6	68.9
Average Interval Between CPAC and SCNECP (months)	144	12	168	84
Liver Biopsy Site	NOS	Right hepatic lobe	NOS	Right hepatic lobe
Average PSA Level (ng/mL) at SCNECP Diagnosis (ref: 0–4)	5.1	0.31	5.12	8.77
Average CEA Level (ng/mL) at SCNECP Diagnosis (ref: 0–4.7 ng/mL)	52.3	4.8	N/A	28.2
Average CA19–9 Level (U/mL) at SCNECP Diagnosis (ref: 0–35 U/mL)	18	1048	N/A	N/A
ADT	Yes	Yes	Yes	Yes
Radiation	Yes	Yes	Yes	Yes
Chemotherapy	Yes	Yes	No	Yes
Radical Prostatectomy	Yes	No	Yes	No
Sites of SCNECP Metastasis	Liver, rectum, various LN	Liver, retroperitoneal LN	Liver, adrenal, retroperitoneal LN, bone	Liver and bone
Average Interval Between SCNECP and Death (months)	26	2	0.75	4.5
**Pathologic Findings**				
Synaptophysin	+	+	+	+
Chromogranin	+	+	+	+
AE1/AE3	+	+	+	+
Ki-67	> 90%	> 90%	80–90%	70–80%
NKX3.1	–	–	Focal +	–
TTF-1	+	–	–	+
PSA	N/A	–	N/A	–
PAP	N/A	–	N/A	+
ERG	+ (weak)*	–	–	–

***SCNECP*** small cell neuroendocrine carcinoma of the prostate, ***CPAC*** conventional prostate adenocarcinoma, ***PSA*** prostate specific antigen, ***NOS*** not otherwise specified, ***CEA*** carcinoembryoninc antigen, ***CA19–9*** carbohydrate antigen 19–9, ***ADT*** androgen deprivation therapy, ***PAP*** prostatic acid phosphatase, ***ERG*** ETS-related gene

*This case was sent out for molecular analysis and the *TMPRSS2-ERG* gene fusion was identified

Briefly, Patient 1 underwent prostatectomy for pT2cN0 CPAC, Gleason 3 + 4 = 7, salvage radiation therapy was administered 2 years later due to elevated PSA (0.ng/mL), then 7 years thereafter PSA was elevated (2.67 ng/mL) with a doubling time of 5.68 months and peripheral androgen blockade was administered with enzalutamide and dutasteride. Subsequently 4 years later, the patient developed right upper quadrant pain for which abdominal ultrasound was initially performed, this demonstrated echogenic lesions that were felt to most likely represent hemangiomas. Shortly thereafter, abdominal MRI was performed, which demonstrated multiple hepatic lesions concerning for metastatic disease. Other sites with metastatic disease identified included the rectum and lymph nodes (pararectal). Following diagnostic liver biopsy, carboplatin and etoposide was administered for 3 months and radioembolization with Yttrium-90 to the right liver was also done. CEA levels rose shortly and 3 months later chemotherapy was started again with cisplatin and irinotecan. Additional chemotherapeutics were attempted and palliative radiation was administered for ongoing pelvic pain and disease progression. The patient ultimately was transitioned to hospice and expired 26 months following the initial diagnosis of SCNEP.

Patient 2 was diagnosed with CPAC (Gleason 4 + 5 = 9) outside the country and underwent radiation treatment for this. Following radiation treatment, PSA became undetectable and goserelin was started. Then, roughly 1 year following radiation treatment, the patient developed right upper quadrant abdominal pain, for which an abdominal CT was performed demonstrating multiple hepatic lesions concerning for metastatic disease. Metastatic disease was also noted in lymph nodes (retroperitoneal). Two weeks later, carboplatin and etoposide was initiated. Disease progression was seen on subsequent imaging studies. Due to the patient’s deteriorating condition and continued disease progression, decision was made to pursue a comfort care approach and the patient expired 2 months following the initial diagnosis of SCNEP.

Patient 3 underwent prostatectomy for pT2cNx CPAC, Gleason 3 + 4 = 7; 7 years later, salvage radiation treatment was administered secondary to biochemical recurrence. Following this, six months later PSA continued to rise (0.57 ng/mL) and androgen deprivation therapy was initiated. The patient was on and off bicalutamide over the course of the next 4 y. Then the patient presented with abdominal distention/pressure roughly two years thereafter. Abdominal CT demonstrated innumerable hepatic metastases. Diffuse osseous metastases, adrenal metastasis, and retroperitoneal lymph node metastases were also seen. Following diagnostic liver biopsy, the decision was made to proceed with a comfort care approach and the patient expired within one month following the initial diagnosis of SCNEP.

Patient 4 presented to the clinic with a markedly elevated PSA (68.9 ng/mL) with bony metastases seen on imaging; on exam his prostate was enlarged and diffusely firm, highly suspicious for malignancy. For presumed metastatic prostate cancer, bicalutamide was immediately initiated. Prostate biopsy was subsequently performed and confirmed CPAC, Gleason 4 + 5 = 9, and one month later leuprorelin was started. Over the course of the next six years, the patient continued on hormonal therapy with palliative radiation given to a humeral bone lesion. Then the patient presented with abdominal pain prompting abdominal CT which showed extensive metastatic disease in the liver and bones. Due to the minimal increase in PSA (5.2 ng/mL) at this time and concern for a second malignancy or evolution of the patient’s prostate cancer, diagnostic liver biopsy was performed. Carboplatin and etoposide was initiated thereafter. Due to disease progression, hospice was initiated and patient expired 4.5 months following the initial diagnosis of SCNEP.

**Fig. 1:**
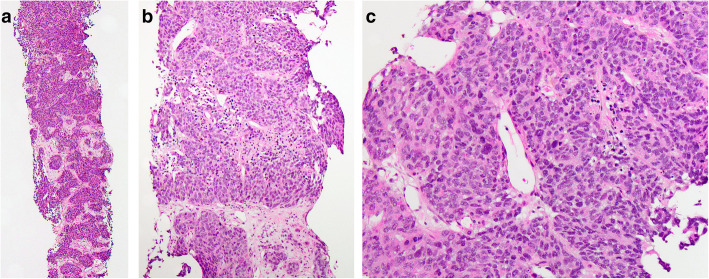
**Liver mass biopsy a** infiltration of liver parenchyma by cells arranged in nests and trabeculae [H&E, 40x], **b** area of central necrosis [H&E, 200x], **c** cells with high N:C, granular chromatin, inconspicuous nucleoli, and frequent mitoses

**Fig. 2:**
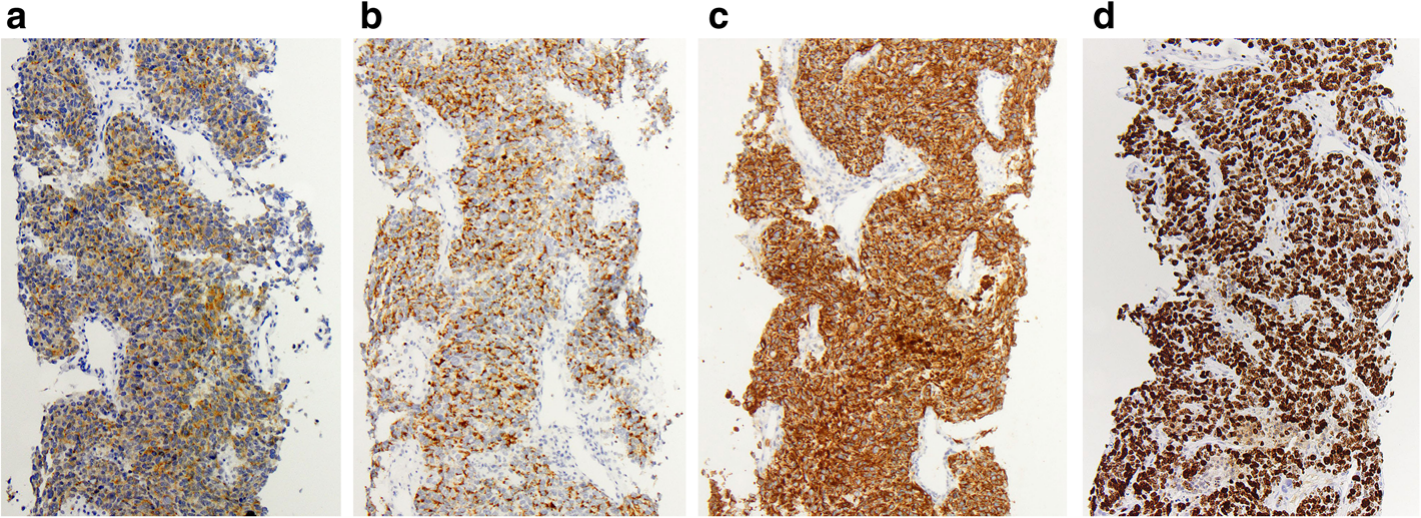
**Immunohistochemistry a** Synaptophysin [100x], **b** Chromogranin [100x], **c** AE1/AE3 [100x], **d** Ki-67 labeling index [100x]

All liver biopsy cases showed similar morphology. At low magnification, the normal hepatic parenchyma was diffusely infiltrated by cells arranged in a nested or trabecular architecture (Fig. 1**a**) with areas of central necrosis (Fig. 1**b**). At higher magnification, the nests were composed of cells with high nuclear: cytoplasmic (N:C) ratios, granular chromatin, inconspicuous nucleoli, and frequent mitoses (Fig. 1**c**). IHC analysis revealed synaptophysin, chromogranin, and AE1/AE3 positivity, with a Ki-67 labeling index of ≥70% of neoplastic cells (Fig. 2**a-d**). NKX3.1 was negative in all but one case, which demonstrated only weak positivity. TTF-1 was positive in two cases (50%). Two cases had prostatic acid phosphatase (PAP) and PSA performed: PSA was negative in both cases, while PAP was positive in one case ERG was weakly positive in one case, this case was also sent out for molecular analysis and the *TMPRSS2-ERG* gene fusion was identified; all other cases were negative for ERG by IHC (Table 1**)**.

## Discussion and conclusions

In contrast to CPAC, neuroendocrine tumors (NETs) of the prostate are rare [1]. Currently, there are five groups of NET outlined by the World Health Organization: (1) Neuroendocrine (NE) cells in usual prostate cancer, (2) adenocarcinoma with Paneth cell-like NE differentiation, (3) well-differentiated NET (carcinoid), (4) small cell NE carcinoma (SCNEC), and (5) large cell NE carcinoma (LCNEC) [5]. Pure SCNECP is very rare, accounting for 0.5–2% of all prostate neoplasms [1]. Most cases of SCNECP are seen in patients with a history CPAC that have been treated with ADT who then develop resistance to therapy [6]. These tumors may also be referred to as castration-resistant prostate cancer [6] and they are associated with an aggressive clinical course, visceral, lytic bony, and pelvic lymph node metastasis, along with low PSA levels [2]. Histologically, around 50% of SCNECP are seen in association with a component of CPAC [7]. In these “mixed” tumors, both components have exhibited TP53 mutations and *TMPRSS2-ERG* rearrangements, as well as loss of Rb protein immunoexpression, suggesting that they are clonal in origin [3]. Here, we present four patients diagnosed with CPAC and managed with ADT, who later developed SCNECP that metastasized to the liver. We discuss the pitfalls when diagnosing metastatic SCNEC and stress the importance of correctly identifying metastatic SCNECP to the liver, as it portends a dismal prognosis.

Identifying the primary site for metastatic SCNEC can be challenging. SCNEC are significantly more common in the lung versus other anatomic sites [8]; however, 10% of extrapulmonary SCNEC occur in the prostate [9]. The liver is a common site of involvement for both metastatic lung [8] and prostate [2] SCNEC, as was seen in our cases. Histologically, SCNEC is characterized by sheet-like, nested, or trabecular architecture with central areas of necrosis. The cells have a high N:C ratio. The nuclei show molding, have a fine “salt-and-pepper” chromatin pattern, and inconspicuous nucleoli. Mitotic figures are often numerous. Regardless of prostate or lung origin, these features are virtually indistinguishable, thus IHC is employed to help determine the primary site. Immunostains that support NE differentiation include chromogranin A and synaptophysin. Traditionally, antibodies to TTF-1 support lung origin, while antibodies to NKX3.1, PSA, or androgen receptor (AR) support prostate origin; however, in terms of SCNECP, expression of AR, PSA [1, 4, 9] and NKX3.1 [3] are often lost. Further, TTF-1 can be positive in 20–80% of SCNEC from any site [8], including SCNECP [1]. Our results support these findings as one case was focally positive for NKX3.1, while the remaining cases were negative, and TTF-1 was positive in 50% of our cases. Although a history of malignancy was not initially provided for these patients, investigation into their medical records revealed both recent and distant histories of CPAC. Additionally, imaging studies did not reveal any lung or urinary bladder lesions. As mentioned previously, studies have demonstrated high specificity of the *TMPRSS2-ERG* gene rearrangement for prostate tumors [3]. Further, the presence of this fusion has been associated with ERG positivity by IHC; however, fluorescence in situ hybridization (FISH) has been shown to be the superior method of detection for SCNECP [10]. This gene rearrangement is seen in ~ 45% of SCNECP a similar incidence seen in CPAC [3, 10]. ERG IHC was performed on all of our cases due to unavailability of the *TMPRSS2-ERG* gene rearrangement by FISH currently at our institution. In one case ERG was found to be weakly positive, all other cases were negative. This weakly positive case was previously sent out for comprehensive molecular analysis and the *TMPRSS2-ERG* gene fusion was identified. Thus, if available the *TMPRSS2-ERG* gene rearrangement can be of utility to confirm prostatic origin, although, its absence does not rule out the possibility of SCNECP; and if this test is not available ERG IHC is also helpful as a supplement [3, 10]. Our results demonstrate that the diagnosis of metastatic SCNECP requires intense correlation of clinical, radiologic, and pathologic findings.

Correct identification of the site of origin for a metastatic SCNEC is significant for patient prognosis and management. Patients with SCNECP are traditionally treated with chemotherapy, particularly cisplatin or etoposide-based therapies [2]; however, surgery, radiation, or immunotherapy may also be considered [9]. Despite these treatment options, there is no standard therapy and the overall survival (OS) remains dismal, with most patients dying ≤1 year after diagnosis [4]. Studies have shown that patients with liver metastasis from CPAC have rapid progression of disease, with one study documenting median survival of four months following the diagnosis of liver metastasis [11]. There are few case reports specifically focusing on the prognosis of SCNECP that has metastasized to the liver, however, these describe a particularly poor prognosis in this setting (collectively, at 25 months all patients died from disease) [12, 13]. In our series, all of our patients succumbed to their disease and the median survival once diagnosed with liver metastasis from SCNECP was 3.25 months. Our findings suggest that patients with liver involvement from metastatic SCNECP have a very poor prognosis. In addition to prognostic information, it is important to correctly diagnose SCNECP as the treatment slightly differs from that of patients with SCNEC of the lung (SCNECL). Standard treatment for these patients is chemotherapy, including cisplatin- or carboplatin-etoposide [14]; however, they may also receive immunotherapy, such as atezolizumab or durvalumab [15]. Although the survival rate for patients with metastatic SCNECL is also dismal, with a 2-year survival rate < 5% [16], it is slightly better than metastatic SCNECP and immunotherapy has been shown to improve survival. Thus, distinguishing the primary site of SCNEC, chiefly between prostate and lung, is vital to patient management.

In conclusion, SCNECP is a rare, aggressive neoplasm that must be considered in the differential diagnosis for metastatic SCNEC, as it is not an infrequent site for extrapulmonary SCNEC. Our case series highlights the importance of considering a prostate primary, even in the setting of normal PSA levels and aberrant IHC profile, when diagnosing a metastatic neuroendocrine carcinoma in the liver. Careful correlation between the patient’s medical history, imaging, and pathology findings is required. Additionally, our results show that once SCNECP metastasizes to the liver, it portends a particularly dismal prognosis (OS: 8.3 months). This emphasizes the need for additional research into treatment modalities for SCNECP.

## Data Availability

Data analyzed above are available from the corresponding author on reasonable request.

## Abbreviations

SCNECP: Small cell neuroendocrine carcinoma of the prostate
CPAC: Conventional prostate adenocarcinoma
ADT: Androgen deprivation therapy
PSA: Prostate specific antigen
IHC: Immunohistochemical
CEA: Carcinoembryonic antigen
CA19–9: Carbohydrate antigen 19–9
PAP: Prostatic acid phosphatase
NET: Neuroendocrine tumor
NE: Neuroendocrine
SCNEC: Small cell NE carcinoma
LCNEC: Large cell NE carcinoma
OS: Overall survival
SCNECL: SCNEC of the lung
